# Retraction: Formation of recombinant bifunctional fusion protein: A newer approach to combine the activities of two enzymes in a single protein

**DOI:** 10.1371/journal.pone.0273534

**Published:** 2022-08-31

**Authors:** The PLOS ONE Editors

The *PLOS ONE* Editors retract this article [1] because it was identified as one of a series of submissions for which we have concerns about authorship, competing interests, and peer review. We regret that the issues were not addressed prior to the article’s publication.

All authors did not agree with the retraction.

## References

[1] Nilpa P, Chintan K, Sayyed RZ, El Enshasy H, El Adawi H, Alhazmi A, et al. (2022) Formation of recombinant bifunctional fusion protein: A newer approach to combine the activities of two enzymes in a single protein. PLoS ONE 17(4): e0265969. 10.1371/journal.pone.0265969 35363796 PMC8975109

